# Book Notices

**Published:** 1869-02

**Authors:** 


### Book Notices.

Transactions of the American Dental Association, at its Fifth and Sixth Annual Meetings, in 1865 and 1866.

This is an octavo volume of over 500 pages, containing a goodly number of addresses, reports, discussions, papers, etc. Among them, we would particularly mention the following:—

The report on Dental Physiology, by Dr. Fitch, of New York, contains suggestions of high importance. An article on Dental Hygiene, by Dr. Chase, of Iowa, is good, but would be better if longer. A paper on the Development and Reproduction of Animal Tissues, by Dr. Buckingham, of Philadelphia, is a good exposition of Virchow's cell theory. The reports upon Pathology and Surgery, by Dr. Atkinson, of New York, contain much that is interesting, but are somewhat transcendental; reminding one of the writings of Andrew Jackson Davis. The remarks on Dental Manipulations, by Dr. McQuillen, of Philadelphia, should be read and remembered by all bunglers. An article on the Sacrifice of Human Teeth, by Dr. Knapp, of New Orleans, ought to be read and studied by all medical juveniles "who love to pull teeth." The remarks on Dental Education, Quack Tooth-Powders, Reproduction of Alveolar Processes, Dental Hygiene, and many other subjects of like import, are all well worthy of perusal. In one of the discussions, the Exsection of Exposed Pulps, by Dr. Allport, of Chicago, is commented upon. The address by the late Dr. Brainard, on Specialties in Medicine, and the remarks on the Brotherhood of Medical Science, by the Editor, are reported in full.

We are glad to see the dentists of this country thus organizing for the promotion of their branch of medical science and practice; and such action betokens that, at no distant day, all dentists of respectable standing must be graduates in medicine.

### SANITARY STATISTICS OF CHICAGO FOR 1868.

The following is a carefully prepared statement of the facts that have been gathered within the jurisdiction of the Chicago Health Department, for the year 1868. These facts pertain to the sanitary and meteorological condition of the city; and there are some interesting facts, which may be discussed by the next social science congress. Although the number of deaths in the city is considerably larger than last year, the city has been considered in a very good hygienic condition throughout the year. The increased mortality may be attributed to the large increase in the rain fall, as well as to the growth of the city.

### OFFICERS OF THE BOARD.

*Commissioners*—Hon. J. B. Rice, President; Samuel Hoard, Wm. Giles, A. B. Reynolds, H. A. Johnson, M.D., J. H. Rauch, M.D., Wm. Wagner, M.D.

*Sanitary Superintendent and Registrar of Vital Statistics*—John H. Rauch, M.D.

*City Physician*—N. T. Quales.

*Health Officer*—Ambrose Burnam.

*Secretary*—Jacob W. Russell.

*Clerks*—H. P. Wright, I. Rosenthal.

| WARD. | *Sanitary Inspectors.* | WARD. | *Sanitary Policemen.* |
|---|---|---|---|
| 1. | E. Powell, M.D. | 1. | W. O. Ludlow. |
| 2. | W. C. Lyman, M.D. | 2. | M. W. White. |
| 3. | J. M. Woodworth, M.D. | 3. | S. Wilson. |
| 4. | E. O. F. Roler, M.D. | 4. | J. W. Buel. |
| 5. | M. Mannheimer, M.D. | 5. | J. Finnucan. |
| 6. | R. M. Lackey, M.D. | 6. | R. Blow. |
| 7. | E. W. Lee, M.D. | 7. | Thos. McGirr. |
| 8. | P. Adolphus, M.D. | 8. | J. J. Heckman. |
| 9. | Geo. Kellogg, M.D. | 9. | H. Musselman. |
| 10. | W. R. Marsh, M.D. | 10. | M. Cole. |
| 11. | T. P. Seeley, M.D. | 11. | L. S. House. |
| 12 | H. M. Lyman, M.D. | 12. | H. L. Alexander. |
| 13. | T. W. Miller, M.D. | 13. | J. Hettinger. |
| 14. | Geo. Schlœtzer, M.D. | 14. | Jas. Keefe. |
| 15. | J. Reid, M.D. | 15. | Geo. Vocke. |
| 16. | D. B. Trimble, M.D. | 16. | G. W. Merrill. |

*Cattle Inspector at Union Stock Yards*—S. P. Hopkins.

*Janitor*—J. Rosenthal.

### MORTALITY FOR THE YEAR 1868.

| Cause | Deaths |
|---|---|
| Abdomen, injury of, | 2 |
| " tumor of, | 7 |
| Abscess, | 5 |
| Accident, drowned | 46 |
| " burned, | 2 |
| " machinery, | 4 |
| " railroad, | 21 |
| " fall, | 7 |
| " gunshot, | 7 |
| " poison, | 8 |
| " scalding | 6 |
| " suffocation | 4 |
| " from other causes, | 31 |
| Anæmia, | 4 |
| Anasarca, | 5 |
| Aneurism, rupture of | 1 |
| Angina, | 13 |
| Anus, cancer of, | 1 |
| Aorta, aneurism of, | 2 |
| Aphthæ, | 5 |
| Apoplexy, | 5 |
| Aseites, | 6 |
| Asphyxia, | 3 |
| Asthma, | 4 |
| Atelectasis pulmonium | 6 |
| Atrophy, | 2 |
| Births, premature, | 170 |
| " still | 395 |
| " tedious, | 9 |
| Biliary calculi, | 1 |
| Bladder, hemorrhage | 1 |
| " inflammation | 1 |
| Bowels, inflammation, | 44 |
| " congestion of, | 6 |
| " cancer of, | 5 |
| " gangrene of, | 1 |
| " hemorrhage of, | 1 |
| " obstruction of | 1 |
| " perforation of | 2 |
| " ulceration of, | 7 |
| Brain, concussion of | 2 |
| Brain, congestion of, | 57 |
| " compression of, | 1 |
| " effusion of, | 1 |
| " disease of, | 16 |
| " inflammation, | 72 |
| " softening of, | 8 |
| " tubercular dise. | 2 |
| Breast, cancer of, | 10 |
| Bronchitis, | 74 |
| Carbuncle, | 2 |
| Cancer, | 7 |
| Calculus, prostrate | 1 |
| Carditis rheumatic, | 1 |
| Catarrh, | 3 |
| Cellutis, | 2 |
| " pelvic, | 2 |
| Cerebellum, disease of | 1 |
| Cerebritis, | 2 |
| Chicken pox, | 2 |
| Childbirth | 10 |
| Chlorosis | 1 |
| Cholera infantum, | 743 |
| " morbus, | 25 |
| Colic, lead, | 1 |
| Colitis, | 1 |
| Convulsions, | 607 |
| " puerperal | 11 |
| Cyanosis, | 6 |
| Croup, | 112 |
| Cystitis, | 1 |
| Debility | 79 |
| Delirium tremens, | 18 |
| Deficient development | 1 |
| Diabetes, | 1 |
| Diarrhœa, | 203 |
| " chronic, | 47 |
| Diphtheria, | 88 |
| Dropsy, | 41 |
| Duodenum, cancer of | 1 |
| Dysentery | 153 |
| Dyspepsia | 2 |
| Eczmea, | 1 |
| Embolia, | 1 |
| Encephaloid, general | 1 |
| Endocarditis | 3 |
| Enteritis, | 39 |
| Enterocolitis, | 16 |
| Epilepsy | 11 |
| Epistaxis, | 1 |
| Erysipelas, | 26 |
| Exhaustion, | 5 |
| Exostosis, | 1 |
| Exposure, | 2 |
| Face, cancer of, | 1 |
| Femur, compound fracture of, | 1 |
| Fever, congestive, | 13 |
| " intermittent, | 7 |
| " puerperal, | 35 |
| " remittent | 9 |
| " scarlet, | 173 |
| " typhoid, | 199 |
| " typhus, | 6 |
| Fungus, hæmatodes, | 2 |
| Gangrene, | 7 |
| Gastritis | 28 |
| Gastro-enteritis, | 13 |
| Gastromalaxia, | 1 |
| Glands, inflammation of parotid sublingual | 2 |
| Glottis, spasms of, | 1 |
| Gout, | 2 |
| Groin, cancer of, | 1 |
| Hemorrhage, | 10 |
| Heart, congestion of, | 1 |
| " disease of, | 80 |
| " dropsy of, | 5 |
| " hypatrophy of, | 5 |
| " inflammation, | 1 |
| Herniplegia, | 5 |
| Hepatitis, | 20 |
| Hernia, | 9 |
| Hydrocephalus, | 129 |
| Hydropericardium, | 2 |
| Hydrothorax, | 14 |
| Ileus, | 3 |
| Inanition, | 97 |
| Indigestion | 6 |
| Infanticide, | 1 |
| Injuries, | 2 |
| Insanity, | 2 |
| Insufficiency of aortic semi-lunar valves, | 1 |
| Intemperance, | 20 |
| Intestines, injuries of, | 1 |
| Ischuria, | 1 |
| Jaundice, | 11 |
| Kidneys, Bright's disease of, | 15 |
| " disease of, | 4 |
| " inflammation | 12 |
| Laryngitis, | 22 |
| Leg, compound fracture of, | 1 |
| Leg, tumor of, | 1 |
| Leucocythæmia, | 1 |
| Lientery, | 1 |
| Liver, abscess of, | 1 |
| " atrophy of, | 2 |
| " cancer of, | 2 |
| " cirrhosis of | 7 |
| " congestion of, | 3 |
| " fatty, degeneration of, | 1 |
| " disease of, | 4 |
| " enlargement of, | 1 |
| " induration of, | 2 |
| " tumor of, | 1 |
| Lungs, abscess of, | 1 |
| " apoplexy of, | 1 |
| " congestion of, | 46 |
| " effusion of, | 1 |
| " emphysema of, | 3 |
| " hemorrhage of, | 11 |
| " paralysis of, | 6 |

| | | | | | |
|---|---|---|---|---|---|
| Malassimilation | 1 | Ovarian tumor, | 5 | Syncope, | 2 |
| Malformation, | 1 | Palate defective, | 2 | Syphilis, | 3 |
| Manslaughter, | 5 | Paralysis, | 21 | Tabes mesenterica, | 158 |
| Measles, | 107 | Paraplegia, | 2 | Tedious labor, | 1 |
| Meningitis, | 66 | Parotitis, | 5 | Teething, | 188 |
| " cerebro-spinal, | 41 | Pericarditis, | 7 | Tetanus, | 12 |
| " tubercular, | 24 | Periostitis, | 1 | Throat, cancer of, | 1 |
| Menses, suppression of | 1 | Peritonæum, cancer of | 1 | " inflammation, | 1 |
| Metral valves, insufficiency of, | 2 | Perdonitis, | 39 | " malformation, | 1 |
| " disease of | 2 | Phargugitis, | 1 | " malignant sore | 1 |
| Metritis | 4 | Phlebites, uterine, | 1 | Tongue, cancer of, | 2 |
| Metro-phlebitis, puerperol, | 1 | Pluenitis, | 4 | Tonsillitis, | 1 |
| Miliasia, puerperum, | 1 | Phthisis pulmonalis, | 418 | Trismus | 1 |
| Miscarriage, | 1 | Pleurisy, | 12 | Tumor, encephaloid, | 1 |
| Morbus coxarius, | 5 | Pneumonia, | 333 | Umbilical cord, sloughing of, | 1 |
| Mouth, canker sore of, | 8 | Purpura, | 1 | Umernia, | 7 |
| " cancer of, | 1 | Pyæmia, | 13 | Urethra, stricture of, | 1 |
| " ulceration of, | 1 | Rectum, cancer of, | 1 | Uterus, cancer of, | 18 |
| Mumps, | 1 | Rheumatism | 7 | " disease, | 1 |
| Myelitis | 4 | Scrofula, | 9 | " hemorrhage of, | 4 |
| Neck, cancer of, | 3 | Scurvy, | 1 | " inflammation | 1 |
| Necrosis, | 1 | Septuernia, | 1 | " rupture of, | 1 |
| Nervous irritation, | 1 | Skull, fracture of, | 4 | " tumor of, | 1 |
| Nutrition, impaired, | 4 | Small-pox, | 146 | Urinary infiltration, | 1 |
| Occipital bone, depression of, | 1 | Spine, caries of, | 3 | Varioloid, | 6 |
| Œsophagus, stricture | 1 | " disease of, | 4 | Vitality, deficient, | 7 |
| Œdema glottedis, | 3 | " dropsy of, | 2 | Whooping-cough | 86 |
| Old age, | 73 | " injury of, | 2 | Worms, | 1 |
| Opium, overdose, | 1 | Stomach, cancer of, | 22 | Unknown, | 22 |
| Ovaritis, | 1 | " congestion of | 3 | Total, | 5960 |
| | | " hemorrhage, | 1 | Accidents & suicides, | 205 |
| | | Stomatitis, | 1 | Immigrants, | 163 |
| | | Suicide, | 33 | | |
| | | Sunstroke, | 23 | | |

AGES.

| | | | | | |
|---|---|---|---|---|---|
| Under 1 | 2238 | 30 to 40 | 447 | 90 to 100 | 2 |
| 1 to 3 | 1187 | 40 to 50 | 323 | 100 to 105 | 2 |
| 3 to 5 | 291 | 50 to 60 | 175 | Unknown | 18 |
| 5 to 10 | 304 | 60 to 70 | 167 | | |
| 10 to 20 | 202 | 70 to 80 | 123 | Total | 5960 |
| 20 to 30 | 447 | 80 to 90 | 34 | | |

| | | | | | |
|---|---|---|---|---|---|
| Males, | 3243 | Females, | 2717 | Total, | 5960 |
| Single, | 4740 | Married, | 1220 | Total, | 5960 |
| White, | 5893 | Colored, | 67 | Total, | 5960 |

NATIVITY.

| | | | | | |
|---|---|---|---|---|---|
| Africa, | 2 | Ireland, | 512 | Sandwich Islands, | 1 |
| Austria, | 6 | Isle of Man, | 2 | Scotland, | 41 |
| Bavaria, | 3 | Italy, | 7 | Sweden, | 137 |
| Bermuda, | 1 | Jamacia, | 1 | Switzerland, | 9 |
| Bohemia | 62 | New Brunswick, | 2 | At Sea, | 2 |
| Canada, | 62 | Newfoundland | 1 | United States, | 920 |
| Chicago | 3162 | Norway | 113 | Wales, | 2 |
| Denmark, | 7 | Nova Scotia, | 2 | West Indies, | 1 |
| England, | 107 | Prince Edward's Island | 1 | Unknown | 29 |
| France, | 13 | Poland, | 10 | | |
| Germany, | 705 | Russia, | 2 | Total, | 5960 |
| Holland | 35 | | | | |

### MORTALITY BY WARDS.

| | | | | | |
|---|---|---|---|---|---|
| 1st Ward, | 92 | Armory, | 2 | Hospital for Women and Children, | 6 |
| 2nd " | 213 | Bridewell, | 9 | Home for Friendless, | 31 |
| 3rd " | 284 | Chicago River, | 15 | Immigrants, | 163 |
| 4th " | 305 | Convent of the Sacred Heart, | 1 | Lake Michigan, | 14 |
| 5th " | 348 | County Jail, | 1 | Protestant Orphan Asylum | 26 |
| 6th " | 356 | Hospital of Alexian Brothers, | 4 | St. Joseph Orphan Asylum, | 28 |
| 7th " | 673 | Hospital of Cook Co., | 126 | Police Station, | 3 |
| 8th " | 371 | Hospital of Lake Co., | 22 | Soldiers' Home, | 7 |
| 9th " | 306 | Marine Hospital | 10 | Yard of Rock Island Railroad, | 1 |
| 10th " | 205 | Mercy Hospital, | 34 | Unknown | 3 |
| 11th " | 336 | St. Luke's Hospital, | 11 | | |
| 12th " | 458 | St. Mary's Hospital, | 1 | | |
| 13th " | 319 | Half-Orphan Asylum, | 2 | | |
| 14th " | 377 | | | | |
| 15th " | 512 | | | | |
| 16th " | 285 | | | Total, | 5960 |

### PERCENTAGE OF DEATHS BY WARDS FOR 1868.

| Wards. | No. of deaths. | Population in 1868. | 1 death in | Wards. | No. of deaths. | Population in 1868. | 1 death in |
|---|---|---|---|---|---|---|---|
| 1st, | 92 | 9,094 | 97¾ | 9th, | 306 | 19,297 | 63 |
| 2d, | 213 | 13,074 | 61⅔ | 10th, | 205 | 12,925 | 63 |
| 3d, | 284 | 15,076 | 60 | 11th, | 336 | 14,340 | 42⅔ |
| 4th, | 305 | 17,796 | 58⅓ | 12th, | 458 | 17,485 | 38 |
| 5th, | 348 | 16,033 | 46 | 13th, | 319 | 11,164 | 35 |
| 6th, | 356 | 13,083 | 6⅔ | 14th, | 377 | 14,839 | 39⅓ |
| 7th, | 673 | 25,492 | 38 | 15th, | 512 | 21,078 | 41 |
| 8th, | 371 | 15,813 | 42½ | 16th, | 285 | 15,465 | 61⅓ |

### METEOROLOGICAL.

Highest and lowest temperature, with range of same, and rain, snow, and mortality for 1868; also mortality for 1867, with rain, showing the influence of want of drainage:—

| 1868. | Highest Temperature. | Lowest Temperature. | Range of Temperature | Rain. | Snow. | Mortality for 1868. | Mortality for 1867. | Rain. |
|---|---|---|---|---|---|---|---|---|
| | de F | de F | | in. | in. | | | in. |
| January, | 41 | 7 | 48 | 1.28 | 7¾ | 441 | 299 | 1.93 |
| February, | 56 | 9 | 65 | 0.93 | 4½ | 425 | 255 | 2.23 |
| March, | 72 | 5 | 67 | 5.25 | 5¾ | 381 | 280 | 1.59 |
| April, | 73 | 19 | 54 | 3.00 | | 307 | 278 | 1.70 |
| May, | 75 | 13 | 62 | 2.73 | | 321 | 241 | 4.22 |
| June, | 90 | 56 | 40 | 3.11 | | 305 | 283 | 1.86 |
| July, | 100 | 70 | 30 | 2.89 | | 897 | 597 | 1.52 |
| August, | 88 | 60 | 28 | 3.58 | | 945 | 697 | 2.33 |
| September, | 84 | 43 | 41 | 7.08 | | 741 | 507 | 0.40 |
| October, | 74 | 37 | 37 | 1.69 | | 448 | 428 | 1.28 |
| November, | 62 | 27 | 35 | 2.60 | 3⅜ | 401 | 370 | 1.77 |
| December, | 48 | 11 | 59 | 1.41 | 8¾ | 348 | 409 | 1.10 |
| Totals, | | | | 35.55 | 30 | 5960 | 4644 | 21.93 |

Meteorological observations made with regard to the temperature at the Dearborn observatory, by Prof. Safford, and at 119 Randolph Street, by J. G. Languth, show a daily difference of 3 deg. F. In 1867 one in 49 died by disease, and in 1868 one in 45¼ of our population. No epidemics have prevailed with the exception of small-pox, in the early part of the year, while there was a marked epidemic tendency during the summer of 1867. The health of the city at this time is remarkably good. An examination of the above table will show that during the months of June, July, August, and September, 16 66-100 inches of rain fell, and that during the same period in 1867 only 6 11-100 inches fell. This, in connection with the great mortality in the wards where there is the least sewerage, also that more than one-half of the total mortality was under five years of age, and 932 more, this than last year; in addition, that the deaths during the months of July, August, and Septemher, when the influence of the want of drainage is most apparent, was nearly as great as the remainder of the year, clearly proves the cause. It may be said that for every inch of rain, that falls over 20 inches during the year, with our limited drainage, costs 100 lives.

### MARRIAGES.

The marriages, as per record of County Clerk were 4684.

### BIRTHS.

The following have been the number of births reported during the year:—

| WARDS. | 1 | 2 | 3 | 4 | 5 | 6 | 7 | 8 | 9 | 10 | 11 | 12 | 13 | 14 | 15 | 16 |
|---|---|---|---|---|---|---|---|---|---|---|---|---|---|---|---|---|
| January, males.. | 3 | 6 | 8 | 18 | 7 | 9 | 30 | 12 | 23 | 15 | 23 | 17 | 5 | 13 | 33 | 12 |
| females | 3 | 11 | 14 | 16 | 3 | 5 | 33 | 14 | 24 | 22 | 20 | 22 | 6 | 13 | 27 | 10 |
| February, males | 5 | 8 | 6 | 15 | 5 | 4 | 38 | 25 | 10 | 13 | 12 | 15 | 4 | 13 | 30 | 18 |
| females | 5 | 6 | 11 | 14 | 2 | 14 | 33 | 15 | 19 | 12 | 9 | 27 | 5 | 9 | 26 | 18 |
| March, males..... | 3 | 7 | 16 | 27 | 9 | 16 | 47 | 25 | 12 | 16 | 14 | 25 | 10 | 22 | 28 | 14 |
| females | 2 | 12 | 12 | 15 | 4 | 12 | 47 | 23 | 25 | 19 | 19 | 24 | 6 | 8 | 16 | 15 |
| April, males...... | 4 | 9 | 13 | 12 | 4 | 10 | 41 | 20 | 24 | 17 | 11 | 23 | 6 | 18 | 29 | 12 |
| females | 3 | 21 | 13 | 17 | 3 | 11 | 34 | 12 | 21 | 4 | 10 | 30 | 6 | 15 | 24 | 15 |
| May, males ...... | 1 | 8 | 7 | 23 | 5 | 9 | 40 | 12 | 21 | 16 | 19 | 29 | 9 | 19 | 20 | 16 |
| females | 3 | 12 | 14 | 20 | 3 | 18 | 32 | 10 | 14 | 11 | 13 | 19 | 7 | 15 | 24 | 17 |
| June, males ....... | 3 | 6 | 12 | 22 | 1 | 15 | 29 | 18 | 18 | 11 | 14 | 33 | 7 | 32 | 20 | 15 |
| females | 2 | 2 | 11 | 24 | 4 | 9 | 35 | 14 | 10 | 12 | 13 | 13 | 4 | 15 | 26 | 5 |
| July, males ...... | 6 | 6 | 10 | 14 | 4 | 15 | 36 | 15 | 33 | 12 | 8 | 19 | 10 | 26 | 33 | 12 |
| females | 3 | 13 | 6 | 17 | 4 | 6 | 25 | 17 | 18 | 15 | 11 | 26 | 8 | 20 | 23 | 11 |
| August, males.... | 6 | 13 | 20 | 19 | 8 | 20 | 43 | 24 | 27 | 14 | 26 | 26 | 13 | 24 | 27 | 13 |
| females | 6 | 19 | 13 | 23 | 8 | 9 | 47 | 19 | 20 | 12 | 19 | 33 | 11 | 28 | 27 | 14 |
| September, males | 6 | 6 | 17 | 17 | 6 | 13 | 38 | 21 | 31 | 17 | 18 | 32 | 11 | 32 | 24 | 9 |
| females | 3 | 7 | 18 | 14 | 6 | 13 | 30 | 19 | 7 | 18 | 14 | 34 | 6 | 19 | 25 | 17 |
| October, males .. | 2 | 13 | 16 | 26 | 9 | 13 | 37 | 19 | 17 | 21 | 16 | 27 | 8 | 21 | 22 | 11 |
| females | 2 | 8 | 16 | 25 | 10 | 14 | 36 | 22 | 23 | 15 | 11 | 25 | 10 | 22 | 31 | 9 |
| November, males | 6 | 15 | 20 | 22 | 8 | 17 | 38 | 18 | 25 | 19 | 15 | 36 | 17 | 27 | 32 | 13 |
| females | 3 | 10 | 9 | 21 | 4 | 14 | 28 | 13 | 22 | 13 | 16 | 32 | 15 | 26 | 23 | 14 |
| December, males | 5 | 9 | 7 | 14 | 5 | 12 | 18 | 13 | 19 | 14 | 16 | 30 | 16 | 23 | 20 | 4 |
| females | 1 | 11 | 15 | 15 | 4 | 12 | 20 | 18 | 18 | 10 | 6 | 13 | 20 | 16 | 16 | 13 |
| Total, .......... | 86 | 235 | 304 | 450 | 126 | 290 | 835 | 418 | 482 | 348 | 363 | 600 | 220 | 476 | 606 | 307 |
| Colored males,.... | 2 | 7 | 10 | | | | | | | 2 | | 3 | | | | |
| females, | 1 | 6 | 7 | | | | | | | 2 | | | | | | |
| By wards, total, | 89 | 248 | 321 | 450 | 126 | 290 | 835 | 418 | 482 | 352 | 363 | 603 | 220 | 476 | 606 | 307 |
| Twins,............ | | 2 | 6 | 8 | 2 | 2 | 9 | 8 | 4 | 2 | 7 | 1 | 1 | 4 | 6 | 6 |
| Triplets,.......... | | | | | 1 | | | | | | | | | | | |

| Months. | Total. | Hospitals. | Colored | Total. | Males. | Females. | Total by months. |
|---|---|---|---|---|---|---|---|
| January, males | 234 | 9 | 1 | 244 | 244 | ..... | 493 |
| females | 243 | 4 | 2 | 249 | ..... | 249 | ..... |
| February, males | 221 | 7 | 4 | 232 | 232 | ..... | 467 |
| females | 225 | 8 | 2 | 235 | ..... | 235 | ..... |
| March, males | 291 | 8 | 1 | 300 | 300 | ..... | 569 |
| females | 259 | 10 | | 269 | ..... | 269 | ..... |
| April, males | 253 | 4 | 2 | 259 | 259 | ..... | 501 |
| females | 239 | 3 | | 242 | ..... | 242 | ..... |
| May, males | 254 | 4 | 3 | 261 | 261 | ..... | 506 |
| females | 232 | 11 | 2 | 245 | ..... | 245 | ..... |
| June, males | 256 | 10 | 2 | 268 | 268 | ..... | 482 |
| females | 199 | 12 | 3 | 214 | ..... | 214 | ..... |
| July, males | 259 | 4 | | 263 | 263 | ..... | 496 |
| females | 223 | 9 | 1 | 233 | ..... | 233 | ..... |
| August, males | 326 | 8 | 3 | 337 | 337 | ..... | 642 |
| females | 292 | 10 | 3 | 305 | ..... | 305 | ..... |
| September, males | 298 | 11 | 2 | 311 | 311 | ..... | 569 |
| females | 250 | 8 | | 258 | ..... | 258 | ..... |
| October, males | 278 | 5 | 3 | 286 | 286 | ..... | 574 |
| females | 279 | 7 | 2 | 288 | ..... | 288 | ..... |
| November, males | 328 | 6 | 2 | 336 | 336 | ..... | 605 |
| females | 263 | 6 | | 269 | ..... | 269 | ..... |
| December, males | 225 | 3 | 3 | 231 | 231 | ..... | 444 |
| females | 208 | 3 | 2 | 213 | ..... | 213 | ..... |
| Total, | | 170 | | | | | 6348 |
| Colored, males | | 1 | | 25 | 25 | | |
| females | | 2 | | 18 | | 18 | |
| By wards, total | | 173 | | | | | |
| Twins, | | 2 | | | | | |

There will be about 150 more reported for the month of December, making the total reported 6498. As near as we can ascertain, we have all, with the exception of about one-fourth, making the total births for the city 8312.

DEATHS BY MONTHS.

| | | | | | |
|---|---|---|---|---|---|
| January, | 441 | June, | 305 | October, | 448 |
| February, | 425 | July, | 897 | November, | 401 |
| March, | 381 | August, | 945 | December, | 348 |
| April, | 307 | September, | 741 | | |
| May, | 321 | | | Total, | 5960 |

ANNUAL REPORT OF THE CITY PHYSICIAN.

CHICAGO, Dec. 31, 1868.—*To the Board of Health of the City of Chicago:* GENTLEMEN—I hereby submit my annual report for the year ending Dec. 31,

SMALL-POX HOSPITAL.

The number of patients remaining in the hospital, Jan. 1, 1868, was 42
Admitted during the year 296

Total, 338

| | |
|---|---|
| The whole number discharged and cured was | 314 |
| The whole number of deaths | 24 |
| Remaining in the hospital at date | none |
| Total, | 338 |

The number and diseases of those discharged cured, were as follows:

| | |
|---|---|
| Vareola confluens | 116 |
| Vareola coherens | 51 |
| Vareola descreta | 98 |
| Varioloid | 41 |
| Rubeola | 6 |
| Scarlatina | 2 |
| Total, | 314 |

The number and diseases of those who died were as follows:

| | |
|---|---|
| Vareola confluens | 20 |
| Phthisis pulmonalis | 3 |
| Valvular disease of the heart | 1 |

The mortality, it will be seen, is rather large, being about 7 per cent. However, when it is considered that four patients died from organic diseases during the stage of convalescence, having had the small-pox in a mild form, and that four died inside of 48 hours after their admission into the hospital, the percentage is considerably reduced and compares favorably, I think, with that of similar institutions elsewhere.

Among those admitted 22 had never been vaccinated, and 19 in which vaccination had been unsuccessful; of the former, 12, and of the latter, 8, proved fatal.

The average time of treatment in the hospital was 27½ days.

The nationalities were as follows:

| | | | | | |
|---|---|---|---|---|---|
| Americans | 123 | Ireland | 22 | France | 4 |
| " colored | 33 | Norway | 21 | Scotland | 5 |
| Germany | 79 | England | 11 | Canada West | 4 |
| Sweden | 29 | Denmark, | 7 | | |
| Total | | | | | 338 |

CITY BRIDEWELL.

The number and diseases of those treated at the City Bridewell during the year ending Dec. 31, 1868, were as follows:

| | | | | | |
|---|---|---|---|---|---|
| Delirium Tremens, | 76 | Pleurisy, | 8 | Wounds | 49 |
| Rheumatism, | 45 | Phthisis pulmonalis, | 12 | Contusions, | 68 |
| Syphilis, | 98 | Neuralgia, | 24 | Sprains, | 14 |
| Gonorrhœa, | 24 | Typhoid fever, | 6 | Abscesses, | 15 |
| Diarrhœa, | 93 | Intermittent fever, | 18 | Dislocations, | 6 |
| Dysentery | 7 | Remittent fever, | 21 | Fractures, | 4 |
| Bronchitis, | 41 | Erysepelas, | 6 | Granular ophthalmia, | 20 |
| Pneumonia, | 6 | Nervous exhaustion | 36 | Iritis, | 10 |
| Total, | | | | | 602 |

The number and diseases of those who died were as follows:

| | | | | | |
|---|---|---|---|---|---|
| Delirium Tremens, | 6 | Erysipelas, | 1 | Phthisis pulmonalis, | 1 |
| Typhoid fever, | 1 | | | | |
| Total, | | | | | 9 |

| | |
|---|---|
| The number sent to the County Hospital was | 19 |
| Sent to the County Poor-House | 16 |
| Sent to the Small-Pox Hospital | 4 |
| Total, | 39 |

The cases sent to the hospital were such as could not conveniently be treated in the institution, with its present accommodations, and those sent to the Poor-House were persons old and infirm, who were in bridewell under the charge of vagrancy.

On the whole, the sanitary condition of the institution during the past year has been good, and no diseases have prevailed excepting those caused by exposure and violence.

All of which is respectfully submitted. N. T. Quales, M.D.,
City Physician.

### SMALL-POX

The following table shows the small-pox cases reported by wards and months:

| WARDS | 1 | 2 | 3 | 4 | 5 | 6 | 7 | 8 | 9 | 10 | 11 | 12 | 13 | 14 | 15 | 16 | Total. |
|---|---|---|---|---|---|---|---|---|---|---|---|---|---|---|---|---|---|
| January, | 4 | 19 | 9 | 9 | 38 | 15 | 29 | 13 | 9 | 11 | 20 | 13 | 2 | 11 | 20 | 6 | 288 |
| February, | 7 | 26 | 18 | 20 | 66 | 10 | 18 | 12 | 11 | 5 | 19 | 4 | 2 | 10 | 12 | 7 | 247 |
| March, | 2 | 12 | 17 | 5 | 35 | 6 | 21 | 9 | 5 | 2 | 19 | 12 | 2 | 11 | 18 | 8 | 184 |
| April, | 3 | 14 | 7 | 14 | 13 | 6 | 11 | 8 | 4 | 3 | 5 | 11 | 3 | 6 | 12 | 10 | 130 |
| May, | 4 | 5 | 2 | 5 | 12 | 9 | 7 | 4 | 3 | 4 | 9 | 4 | 15 | 7 | 16 | 4 | 110 |
| June, | 7 | 9 | 7 | 1 | 5 | 1 | ... | ... | 2 | 1 | 4 | 2 | 2 | 3 | 6 | 4 | 54 |
| July, | 2 | ... | ... | 1 | ... | ... | ... | 1 | ... | 3 | 2 | 1 | 1 | 2 | 3 | ... | 16 |
| August, | ... | ... | ... | 1 | ... | ... | ... | ... | ... | ... | ... | ... | ... | 2 | ... | ... | 3 |
| September, | ... | ... | ... | ... | ... | ... | ... | ... | ... | ... | ... | ... | ... | 1 | 3 | ... | 4 |
| October, | ... | ... | 1 | ... | ... | ... | 7 | ... | ... | 1 | ... | ... | ... | ... | 1 | ... | 10 |
| November, | ... | 1 | ... | ... | 1 | ... | ... | ... | ... | ... | 1 | ... | 2 | 1 | 7 | ... | 13 |
| December, | ... | ... | ... | ... | ... | ... | 2 | 1 | ... | 2 | ... | ... | 4 | 1 | ... | ... | 10 |
| Total, | 29 | 36 | 61 | 56 | 170 | 47 | 95 | 48 | 37 | 29 | 79 | 47 | 33 | 55 | 98 | 39 | 1009 |

### VARIOLOID.

The following table will show the varioloid cases reported by wards and months:

| WARDS | 1 | 2 | 3 | 4 | 5 | 6 | 7 | 8 | 9 | 10 | 11 | 12 | 13 | 14 | 15 | 16 | Total. |
|---|---|---|---|---|---|---|---|---|---|---|---|---|---|---|---|---|---|
| January, | 3 | 12 | ... | 6 | 19 | 5 | 11 | 4 | 3 | 3 | 6 | 2 | ... | 9 | 2 | 4 | 89 |
| February, | 3 | 3 | 4 | 10 | ... | 3 | 8 | 6 | 5 | ... | 5 | 2 | ... | 3 | 2 | 3 | 57 |
| March, | 6 | 1 | 3 | 3 | 8 | 7 | 3 | 7 | 1 | 1 | 3 | 1 | ... | 1 | 1 | 1 | 48 |
| April, | ... | 4 | 1 | 2 | 6 | 3 | 6 | 2 | 3 | 4 | 2 | 1 | 1 | 4 | 2 | 5 | 48 |
| May, | 1 | 4 | 2 | 1 | 3 | 1 | 4 | ... | 1 | 1 | ... | ... | 3 | 3 | 1 | 1 | 24 |
| June, | ... | ... | 3 | 2 | ... | ... | 1 | ... | 1 | ... | 1 | 2 | 1 | ... | 4 | ... | 15 |
| July, | ... | ... | ... | ... | ... | ... | ... | 1 | ... | ... | ... | ... | 1 | ... | ... | ... | 2 |
| August, | ... | ... | ... | ... | ... | ... | ... | ... | ... | ... | ... | ... | ... | ... | ... | ... | ... |
| September, | ... | ... | ... | ... | ... | ... | ... | ... | ... | ... | ... | ... | ... | ... | ... | ... | ... |
| October, | ... | ... | ... | ... | ... | ... | ... | ... | ... | ... | ... | ... | ... | ... | ... | ... | ... |
| November, | ... | 1 | ... | 1 | ... | ... | 1 | ... | ... | ... | ... | 1 | ... | 1 | 1 | 2 | 8 |
| December, | ... | ... | ... | ... | ... | ... | ... | ... | ... | ... | ... | ... | ... | 2 | ... | ... | 2 |
| Total, | 13 | 25 | 13 | 25 | 36 | 19 | 34 | 20 | 14 | 9 | 17 | 9 | 6 | 23 | 13 | 16 | 292 |

Total of small-pox and varioloid cases, 1301

### NUMBER OF PATIENTS REMOVED TO THE SMALL-POX HOSPITAL.

| | | | | | |
|---|---|---|---|---|---|
| January | 46 | June | 30 | October | 8 |
| February | 64 | July | 4 | November | 16 |
| March | 46 | August | 3 | December | 2 |
| April | 36 | September | 2 | | |
| May | 41 | | | Total, | 298 |

### GRATUITOUS VACCINATIONS FOR 1868.

| | | |
|---|---|---|
| Dr. E. Powell ......... 34 | Dr. P. Adolphus .........670 | Dr. Geo. Schloetzer.....187 |
| Dr. W. C. Lyman .......168 | Dr. Geo. Kellogg ...... 35 | Dr. J. Reid ..............170 |
| Dr. J. M. Woodworth 100 | Dr. W. R. Mulsh ......350 | Dr. D. B. Trimble...... 80 |
| Dr. E. O. F. Roler ..... 90 | Dr. T. P. Seeley ........100 | Dr. N. T. Quales ........313 |
| Dr. M. Mannheimer....300 | Dr. H. M. Lyman.......150 | Sanitary superinten'nt 278 |
| Dr. R. M. Lackey.......260 | Dr. T. W. Miller........125 | |
| Dr. E. W. Lee ...........600 | | Total, ............... 4010 |

During the year, 10,500 children were examined in private schools, and 4700 in public schools. Eight hundred and ten vaccine crusts were distributed.

### INSPECTION OF IMMIGRANT TRAINS.

Number of immigrant trains inspected by sanitary inspectors and sanitary policemen of First, Second, Third, and Eleventh wards, were 210.

### NUMBER OF IMMIGRANTS ARRIVED.

| | |
|---|---|
| By Michigan Central railroad | 26,873 |
| By Michigan Southern railroad | 24,500 |
| By all other routes | 26,000 |
| Total | 77,373 |

Number of immigrants, suffering with small-pox, removed from depots to small-pox hospital, were 48.

### MEAT INSPECTION.

All meat and vegetable markets in the city are under constant inspection. During the year there were condemned:—330 carcasses of mutton; 35 carcasses of veal; 23 carcasses of pork; 16 carcasses of beef.

Cattle inspected at Union stock yards, from August 30th to December 31st, 1868.

### INSPECTION OF CATTLE.

| | | |
|---|---|---|
| August.................. 4,353 | October ............... 34,779 | December ............ 21,231 |
| September........... 30,383 | November ........... 29,650 | |
| | | Total ............ 120,396 |

Of these, 178 were condemned.

Three hundred and twenty cows, and many sows, with young, were prevented from being sold for consumption in this market.

No record of the sheep and hogs that were kept out of the market.

### CATTLE INSPECTION AT SLAUGHTER-HOUSES.

In order to prevent the introduction of dressed meat into the market, during the prevalence of the "cattle disease," the internal organs of 16,800 cattle were examined. A record was kept of the weights of the livers and spleen of 9000 of these.

Seventy-three cattle, suffering from the Texas fever, were condemned.

Sixty-one cattle, injured and unfit for food, were condemned.

*Post mortem* examinations were made of 142 cattle that died of Texas fever.

---

Death from Chloroform.—Dr. Van Buskirk, of Gorham, Ohio, was found dead in his bed a short time ago. His death was supposed to have been caused by the inhalation of chloroform when alone, to relieve a nervous headache.—*N. Y. Med. Record.*

### Book Notices.

From Lindsay & Blakiston, Publishers, we have received, through the agency of S. C. Griggs & Co., of this city, the following works:—

The Physician's Dose and Symptom Book: Containing the Doses and Uses of all the Principal Articles of the Materia Medica, and Officinal Preparations; also, a Table of Weights and Measures; Rules to Proportion the Doses of Medicines; Common Abbreviations Used in Writing Prescriptions; Table of Poisons and Antidotes; Classification of the Materia Medica; Pharmaceutical Arrangement; Table of Symptomatology; Outlines of General Pathology and Therapeutics. By JOSEPH M. WYTHES, A.M., M.D., Author of the "Microscopist," etc., etc. Eighth Edition. Philadelphia: Lindsay & Blakiston. 1868. Price $1.

The whole matter indicated by the above copious title is enclosed in a small duodecimo volume of 263 pages; designed for convenience as a pocket companion.

---

Pronouncing Medical Lexicon: Containing the Current Pronunciation and Definition of the Terms Used in Medicine and the Collateral Sciences. With Addendum: Containing the Abbreviations Used in Prescriptions, and List of Poisons and their Antidotes. By C. H. CLEVELAND, M.D. Philadelphia: Lindsay & Blakiston. 1869. Price $1.25.

This is the eleventh edition of the well known Cleveland's Pocket Dictionary; a duodecimo of 302 pages.

---

The Use of the Laryngoscope in Diseases of the Throat. With an Essay on Hoarseness, Loss of Voice, and Stridulous Breathing, in Relation to Nervo Muscular Affections of the Larynx. By MERRILL MCKENZIE, M.D., London; M.P.C.P.; Physician to the Hospital for Diseases of the Throat; and Assistant-Physician, and Co-Lecturer on Physiology, at the London Hospital. Second Edition. With Additions, and a Chapter on the Examinations of the Nasal Passages, by J. SOLIS COHEN, M.D., Author of "Inhalation: its Therapeutics and Practice," etc. With 2 Lithograph Plates and 51 Illustrations on Wood. Philadelphia: Lindsay & Blakiston. 1869. Price $3.00.

This is an elegantly published volume, octavo, of 289 pages; the paper, type, and illustrations, are all excellent. It is a book that should be in every physician's library.

---

On Chronic Bronchitis, especially as Connected with Gout, Emphysema, and Diseases of the Heart: Being Clinical Lectures delivered at the Middlesex Hospital. By E. HEADLAW GREENHOWE, M.D.; Fellow of Royal College of Physicians; Consulting Physician to the Western General Dispensary; Senior Assistant-Physician to the Middlesex Hospital. Philadelphia: Lindsay & Blakiston. 1869. Price $2.25.

This volume embraces eight Lectures on topics of great practical interest and importance. The first is devoted to a general consideration of the Etiology of Bronchitis, and its Relation to other Diseases; the second, to Bronchitis, from Mechanical Irritation; the third and fourth, to Gouty Bronchitis; the fifth and sixth, to Emphysema, and the Connection of Bronchitis with it; the seventh and eighth, to Bronchitis as Connected with the various Organic Diseases of the Heart. These several lectures are illustrated by over 40 clinical cases. The work is a valuable addition to the literature of bronchitis.

---

We have received from the publishing house of Williams Wood & Co., of New York, through the hands of S. C. Griggs & Co., of this city, the following works:—

Pathological Anatomy of the Female Sexual Organs. By JULIUS M. KLOB, M.D., Professor at the University of Vienna. Translated from the German, by JOSEPH KAMMERER, M.D., Physician to the German Hospital and Dispensary, New York, and BENJAMIN F. DAWSON, M.D., Assistant to the Chair of Obstetrics, in the College of Physicians and Surgeons, New York. New York: Williams Wood & Co., Publishers. 1868. 299 pages. Price $3.50.

This is a very valuable work, on a highly interesting subject. The translators and American publishers have executed their tasks in good style, and deserve the thanks of the profession.

